# Author Correction: Organ-specific genome diversity of replication-competent SARS-CoV-2

**DOI:** 10.1038/s41467-022-33970-x

**Published:** 2022-10-21

**Authors:** Jolien Van Cleemput, Willem van Snippenberg, Laurens Lambrechts, Amélie Dendooven, Valentino D’Onofrio, Liesbeth Couck, Wim Trypsteen, Jan Vanrusselt, Sebastiaan Theuns, Nick Vereecke, Thierry P. P. van den Bosch, Martin Lammens, Ann Driessen, Ruth Achten, Ken R. Bracke, Wim Van den Broeck, Jan Von der Thüsen, Hans Nauwynck, Jo Van Dorpe, Sarah Gerlo, Piet Maes, Janneke Cox, Linos Vandekerckhove

**Affiliations:** 1 HIV Cure Research Center, Department of Internal Medicine and Pediatrics, Ghent University Hospital, Ghent University, Ghent, Belgium; 2 BioBix, Department of Data Analysis and Mathematical Modelling, Faculty of Bioscience Engineering, Ghent University, Ghent, Belgium; 3 Department of Pathology, Ghent University Hospital, Ghent University, Ghent, Belgium; 4 Department of Pathology, Antwerp University Hospital, Edegem, Belgium; 5 Faculty of Medicine and Health Sciences, University of Antwerp, Wilrijk, Belgium; 6 Faculty of Medicine and Life Sciences, Hasselt University, Hasselt, Belgium; 7 Department of Infectious Diseases and Immunity, Jessa Hospital, Hasselt, Belgium; 8 Department of Morphology, Faculty of Veterinary Medicine, Ghent University, Merelbeke, Belgium; 9 Department of Radiology, Jessa hospital, Hasselt, Belgium; 10 PathoSense BV, Lier, Belgium; 11 Department of Virology, Parasitology and Immunology, Faculty of Veterinary Medicine, Ghent University, Merelbeke, Belgium; 12 Department of Pathology, Erasmus MC, Rotterdam, The Netherlands; 13 Department of Pathology, Jessa hospital, Hasselt, Belgium; 14 Laboratory for Translational Research in Obstructive Pulmonary Diseases, Department of Respiratory Medicine, Ghent University Hospital, Ghent University, Ghent, Belgium; 15 Department of Biomolecular Medicine, Ghent University, Ghent, Belgium; 16 Laboratory of Clinical and Epidemiological Virology, Department of Microbiology, Immunology and Transplantation, Rega Institute, KU Leuven, Leuven, Belgium

**Keywords:** SARS-CoV-2, Viral pathogenesis, Clinical microbiology

Correction to: *Nature Communications* 10.1038/s41467-021-26884-7, published online 16 November 2021

The original version of this Article contained an error in ‘Virion pelleting from plasma’ in Methods, which incorrectly read ‘One microliter of plasma (citrate tubes) was diluted in 10 mL of …’. The correct version states ‘milliliter’ in place of ‘microliter’. This has been corrected in both the PDF and HTML versions of the Article.

